# Comparison of functional metacarpal splint and ulnar gutter splint in the treatment of fifth metacarpal neck fractures: a prospective comparative study

**DOI:** 10.1186/s12891-019-2556-6

**Published:** 2019-04-13

**Authors:** Gokhan Kaynak, Huseyin Botanlioglu, Mustafa Caliskan, Bedri Karaismailoglu, Mahmut Kursat Ozsahin, Soner Kocak, Enis Yildirim, Onder Aydingoz, Mehmet Fatih Guven

**Affiliations:** 1 Department of Orthopaedics and Traumatology, Istanbul University – Cerrahpasa, Cerrahpasa Medical Faculty, Kocamustafapasa Cad. No: 53, Fatih, Istanbul, Turkey; 2 Department of Orthopaedics and Traumatology, Istanbul Kanuni Sultan Suleyman Training and Research Hospital, Istanbul, Turkey; 3 Department of Orthopaedics and Traumatology, Ayancik State Hospital, Sinop, Turkey; 4 Department of Orthopaedics and Traumatology, Konak Hospital, Sakarya, Turkey

**Keywords:** Fifth metacarpal neck fracture, Metacarpal functional splint, Ulnar gutter splint, Grip strength, Metacarpal shortening, Angulation

## Abstract

**Background:**

Fifth metacarpal fractures are the most common fractures of the hand. These fractures are generally treated with conservative methods. The aim of this study was to compare the radiological and clinical outcomes of two conservative treatment methods, functional metacarpal splint(FMS) and ulnar gutter splint(UGS), for the treatment of fifth metacarpal neck fractures.

**Methods:**

A prospective comparative study was designed to assess the conservative treatment of isolated and closed stable fractures of the fifth metacarpal neck. In total, 58 patients were included in the study and were treated with FMS or UGS after fracture reduction in a consecutive order. Angulation, shortening and functional outcome (*Quick*DASH scores and grip strengths) were evaluated at the 2nd and 6th months.

**Results:**

Forty patients returned for follow-up. Twenty-two patients were treated with FMS, and 18 patients were treated with UGS. The average age was 28 years (SD ± 12, range;18–43) in the FMS group and 30 years (SD ± 14, range;18–58) in the UGS group. After reduction, significant correction was achieved in both groups, but the average angulation was lower in the FMS group(16 ± 7) compared with the UGS group (21 ± 8)(*p* = 0.043). However, this better initial reduction in FMS group(16 ± 7) could not be maintained in the 1st month follow-up (21 ± 5) (*p* = 0.009). In the FMS group, the improvement in *Quick*DASH scores between the 2nd and 6th month follow-up was significant (*p* = 0.003) but not in the UGS group(*p* = 0.075). When the expected grip strengths were calculated, the FMS group reached the expected strength values at the 2nd month follow-up, whereas the UGS group still exhibited significantly lower grip strength at the 2nd month follow-up(*p* = 0.008). However, at the end of the 6th month follow-up, both groups exhibited similar reduction, *Quick*DASH and grip strength values.

**Conclusions:**

In stable 5th metacarpal neck fractures, FMS is adequate to prevent loss of reduction and yields faster improvement in clinical scores with earlier gain of normal grip strength compared with UGS. However, in the long term, both FMS and UGS methods yield similar radiological and clinical outcomes. Patient comfort and compliance may be better with FMS due to less joint restriction, and these findings should be considered when deciding the treatment method.

**Trial registration:**

ISRCTN79534571

The date of registration: 01/04/2019

**Type of study/level of evidence:** Therapeutic, II.

## Background

Hand fractures are the most common fractures of the body, and 18–44% of all fractures in the hand occur in the metacarpal bones [1–4]. Fractures of the fifth metacarpal are the most common fractures in the hand [2], and most of them are treated conservatively via ulnar gutter splint (UGS) [5]. Closed treatment of these fractures with immobilization can lead to complications, such as malunion, extension lag, stiffness and reduced grip strength [5]. Although satisfactory clinical results were reported with the application of the traditional UGS splint, it may lead to high patient discomfort due to the limitation of wrist and finger movements [5]. Different functional splinting and soft wrap/bandage treatment methods were described to overcome the patient discomfort and the majority of the studies reported satisfactory outcomes [6–8]. Acceptable outcomes have also been reported after the treatment of distal and diaphyseal fractures of the fifth metacarpals with a well-molded functional metacarpal splint (FMS), which is less restricting and more comfortable compared with UGS [5, 9]. However, the evidence for its superiority in terms of reduction maintenance and functional outcome is still lacking.

The aim of this study is to compare UGS and FMS in the treatment of fifth metacarpal neck fractures regarding the functional and radiological results to understand whether UGS represents an overtreatment of fifth metacarpal neck fractures that are suitable for conservative management. We hypothesized that in the treatment of stable metacarpal neck fractures, FMS is adequate to prevent loss of reduction and results in a faster recovery compared with UGS.

## Methods

Between 2012 and 2017, a prospective comparative study was designed for the treatment of 5th metacarpal neck fractures. Patients were recruited from the University Hospital Emergency Room (E.R.) and were treated with FMS or UGS. The power analysis, based on previous reports [10], revealed that at least 14 subjects were needed in both groups for a power of 0.8 at a significance level of 0.05. The treatment plan was applied to patients in a consecutive manner based on referral time (quasi-randomisation). The orthopedist making the intervention at the E.R. checked the last treatment performed and applied the other choice of treatment. Patients with isolated and closed neck fractures of the fifth metacarpal with no rotational deformity or associated injury between age 18 and 60 years were included in the study. Unstable, open, comminuted and intraarticular metacarpal neck fractures were not included in the study. In total, 58 patients who meet these criteria were treated and included in the study. Eighteen patients were lost to follow-up and excluded from the study; thus, 40 patients were included in the final evaluation. The local ethics committee approved the study, and informed consent was obtained from all patients.

An acceptable angulation value was set as 40° for the 5th metacarpal neck in 30° oblique views after closed reduction and splinting [11]. In fact, the actual amount of angulation can be determined with lateral radiographies. However, due to the measuring difficulty related to superposition and to prevent intraobserver variability in measurements, we preferred to use oblique radiographies that were acquired in pronation. Malrotation was assessed clinically compared with the contralateral side by lining up the fingernail in partial flexion or full flexion if possible. Although the literature is inconsistent about the benefit of the reduction, due to some aesthetic and functional concerns proposed by some authors [11–13], we applied the Jahss maneuver [14] (flexing the MP and PIP joints to 90 degrees and using the proximal phalanx to push up the metacarpal head) in all patients to obtain a reduction close to anatomical alignment [15]. We were able to achieve an acceptable reduction for all patients in the first or second attempt.

**Fig. 1:**
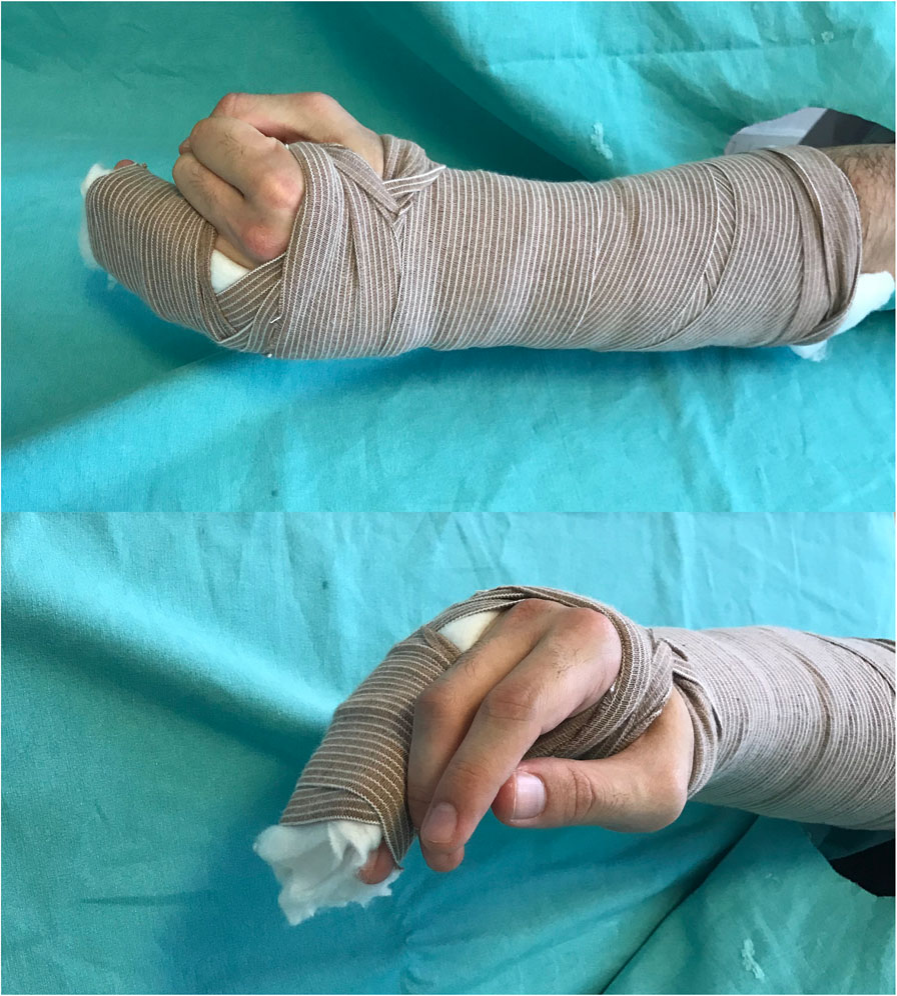
Ulnar gutter splint

**Fig. 2:**
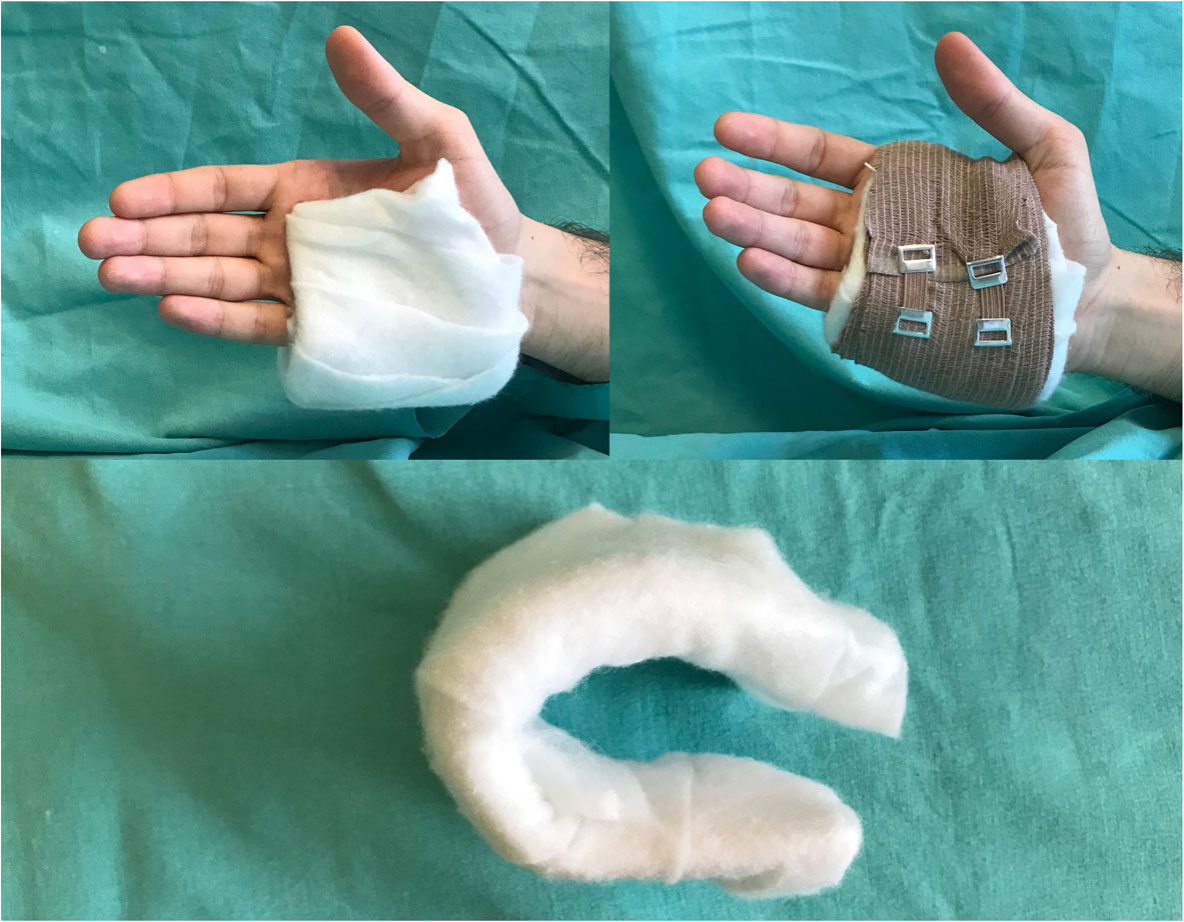
Functional metacarpal splint

After fracture reduction, UGS was applied from the medial side of the hand and the forearm with the wrist at 30° extension, the metacarpophalangeal (MCP) joint at 70° flexion and the proximal interphalangeal (PIP) joint at full extension. Slight pressure was applied over the splint to distal volar and proximal dorsal sides of the fracture to prevent reduction loss (Fig. 1). FMS was placed according to the 3-point principle (one dorsal, two volar). The dorsal contact point was over the fracture site. One volar contact point was located over the metacarpal head, and one volar contact point was located over the metacarpal shaft. FMS does not limit the motion of MCP and wrist joints. The splint length was similar to metacarpal length (Fig. 2).

**Fig. 3:**
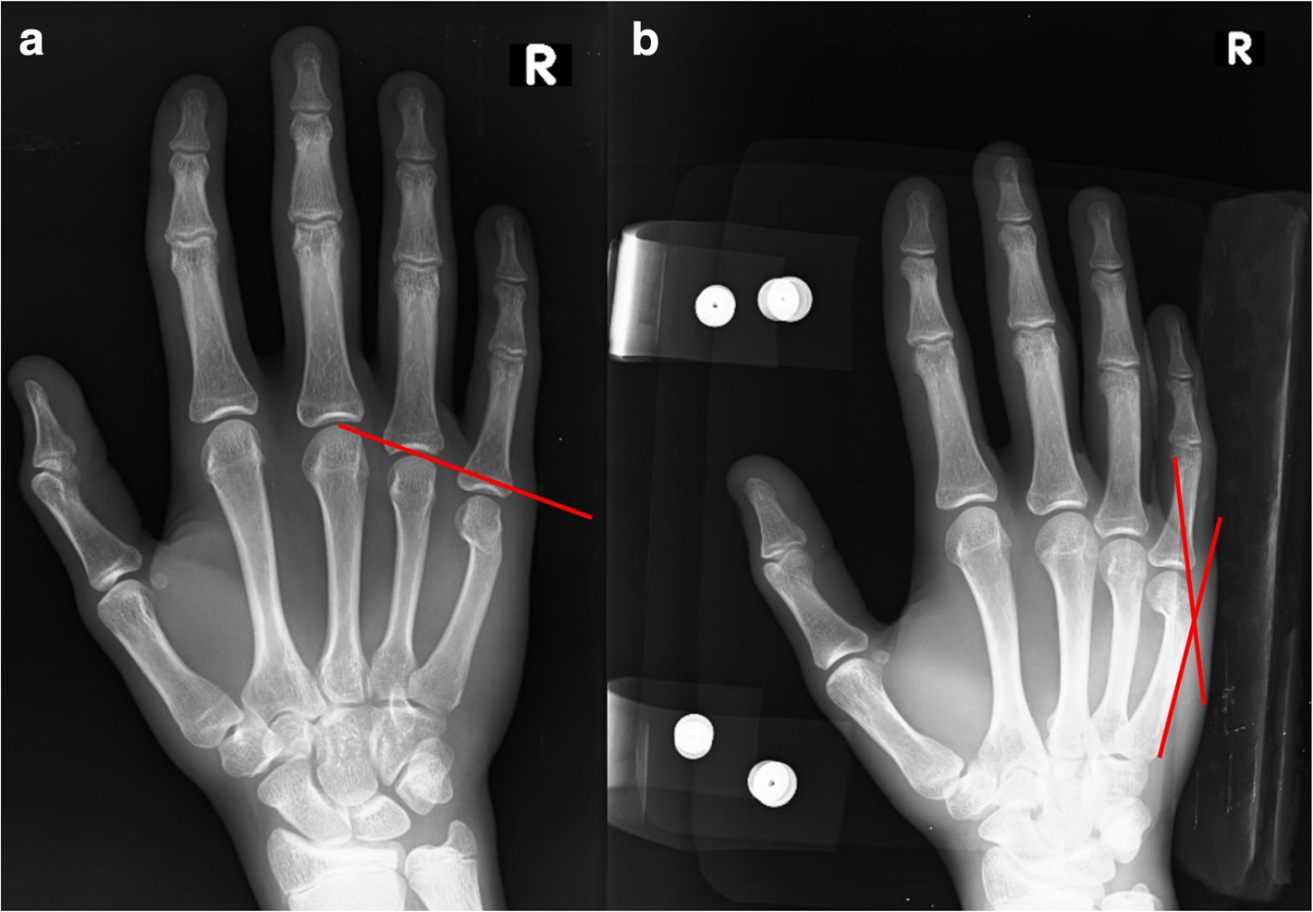
**a** Method of Shortening Stipulated (SH – Stip), which is used for the measurement of shortening on AP radiography. A line was drawn through the most distal point of the heads of the neighboring 3th and 4th metacarpals. The shortening was defined as the distance from this line to the most distal point of the fractured fifth metacarpal [16]. **b** Method of Dorsal Cortex – oblique (DC – 30), which is used for the measurement of angulation at a 30° oblique view. The measurement lines were drawn at the most dorsal part of the metacarpal cortices [16]

**Fig. 4:**
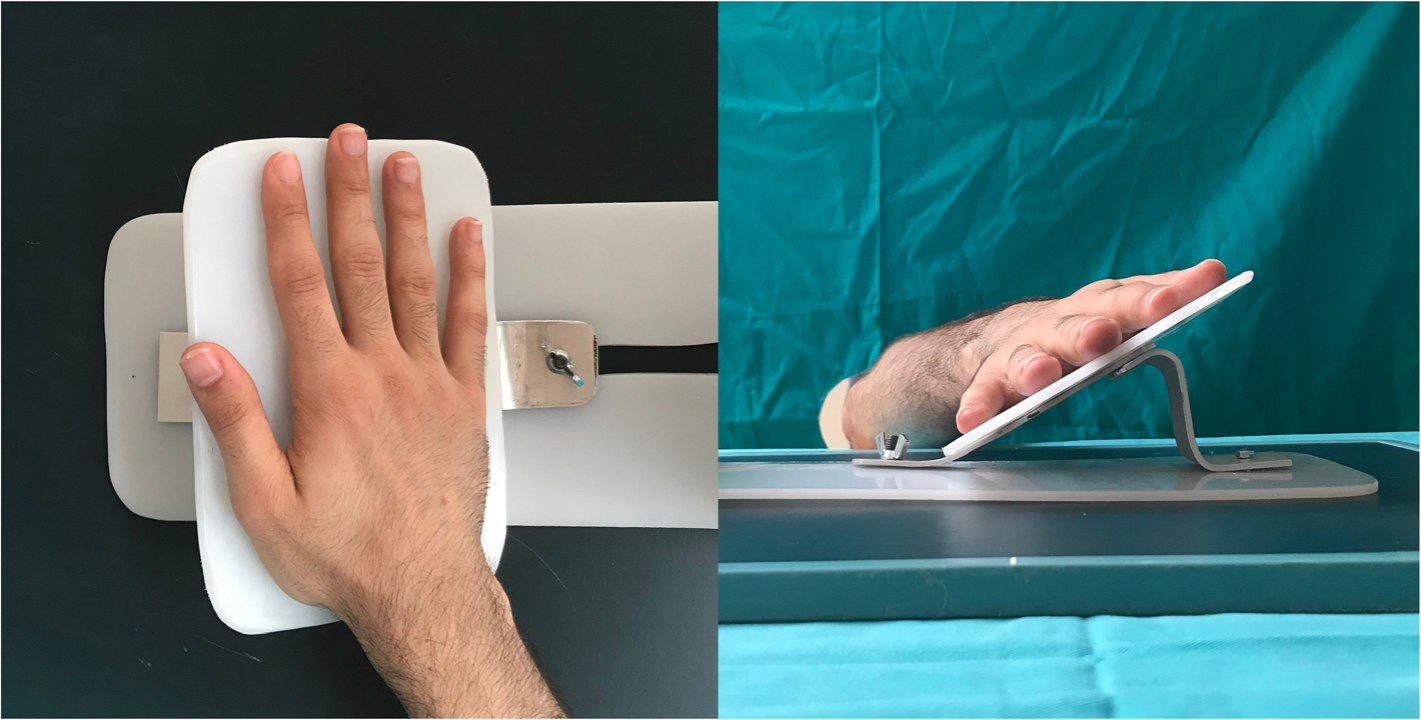
The device used to posture the hand to obtain a standardized 30° oblique view

All patients were asked to attend the following follow-up visits: 1 week, 1 month, 2 months and 6 months after injury. Anteroposterior (AP) and 30° oblique X-rays were obtained following fracture reduction with a splint and at the first and sixth months without the splint. Angulation was measured on 30° oblique views by setting the upper cortex as a reference point [16] (Fig. 3a). Shortening was measured on AP view using the Shortening Stipulated (SH-Stip) technique [16] (Fig. 3b). A device was used to posture the hand to obtain a standardized 30° oblique view (Fig. 4). At the first month follow-up, patients’ splints were removed after the clinical assessment of fracture healing and the detection of callus formation on direct radiographies. After splint removal, all patients were allowed to use their hand, and grip strength exercises with a malleable stress ball were initiated for all patients. The age, gender, hand dominance, injury mechanism, and number of reduction attempts were documented for each patient (Table 1).

**Table 1:** Distribution of descriptive properties

		UGS (*n* = 18)	FMS (*n* = 22)	*P*
Mean Age		27.8	30.2	^a^0.61
Gender	Female (2.5%)	1	0	^b^0.74
	Male (97.5%)	17	22	
Fracture Mechanism	Punching a hard object (75%)	14	16	^c^0.71
	Fall (25%)	4	6	
Reduction Attempts	One (87.5%)	16	19	^c^0.82
	Two (12.5%)	2	3	
Injury Side	Dominant (85%)	15	19	^c^0.79
	Non-dominant (15%)	3	3	

*UGS* Ulnar gutter splint, *(FMS)* Functional metacarpal splint

^a^Mann Whitney U test

^b^Fisher’s Exact test

^c^Pearson Chi – Square test

Functional outcomes were measured with the shorter version of Disabilities of the Arm, Shoulder, and Hand (*Quick*DASH) Questionnaire [17, 18]. The validated Turkish version of the QuickDASH questionnaire was used [19]. Grip strength was tested by Jamar Plus*+* hand dynamometer (Patterson Medical, Illinois, USA). It is a device providing accurate results, used and recommeded by previous studies [20, 21]. Test was applied to both hands in neutral position to minimize the effect of other factors. Both hands were tested thrice at each evaluation, and the mean value of three results was calculated. Loss of grip strength was also determined by measuring the difference in mean strength value between the involved and noninvolved extremities. The evaluation was made on the basis of the assumption that the average hand grip strength for the dominant hand is 10% greater than the nondominant hand, and expected grip strengths of the hands were calculated accordingly [22]. *Quick*DASH and grip strength tests were applied at the first month after splint removal (second month evaluation) and sixth months after trauma. All radiological and clinical evaluations were made by the same orthopedist who was blinded to the study.

The NCSS (Number Cruncher Statistical System) 2007 (Kaysville, Utah, USA) program was used for statistical analysis. Mean, standard deviation (SD), median, frequency, ratio, and minimum and maximum values were used as descriptive statistical methods. Student t test was used for two-group comparisons of quantitative data with a normal distribution, and Mann-Whitney U test was used for two-group comparisons of data without a normal distribution. Paired sample t-test was used for intragroup comparison of parameters with a normal distribution, and a Wilcoxon Signed Rank test was used for intragroup comparison of parameters without a normal distribution. In the analysis of follow-up values with a normal distribution, two-way ANOVA test was used to evaluate the variables, and the Bonferroni test was used to evaluate binary comparisons. *P* values were multiplied by six for Bonferroni correction. In the analysis of follow-up values without a normal distribution, the Wilcoxon Signed Rank test was used to evaluate the variables, and the Friedman test was used to evaluate binary comparisons. Statistical significance was set as *p* < 0.05.

## Results

Among 40 patients, 22 patients (55%) were treated with FMS, and 18 patients (45%) were treated with UGS. The average age was 28 years (SD ± 12, range; 18–43) in the FMS group and 30 (SD ± 14, range; 18–58) in the UGS group. In total, 86% (19) of patients in the FMS group and 83% (15) of patients in the UGS group injured their dominant hand. All patients provided a history of normal function and full motion before the fracture. No statistically significant differences in gender, age, hand dominance, injury mechanism and number of reduction attempts were noted between the two groups. The distributions of descriptive characteristics are presented in Table 1.

When the fracture angulation of both groups was analyzed, the average angulation of the fractures was similar between the groups before reduction. After reduction, significant correction was achieved in both groups, but the average angulation was lower in the FMS group (16 ± 7) compared with the UGS group (21 ± 8). (*p* = 0.043) In the FMS group, the initial reduction (16 ± 7), which was better compared with the UGS group, could not be maintained in the 1st month follow-up (21 ± 5). (*p* = 0.009) However, the reduction at the 1st month was maintained at the 6th month follow-up in the FMS and UGS groups (Table 2).

**Table 2:** Evaluation of periodic angular measurements and differences based on the splint method

		UGS (*n* = 18)	FMS (*n* = 22)	*P*
Angulation Measurements				
Fracture	Min-Max (Median)	14–62 (33)	15–50 (31,5)	^a^0.710
	Mean ± SD	32.11 ± 11.51	30.82 ± 10.29	
Initial reduction	Min-Max (Median)	4–30 (22,5)	7–30 (14)	^a^0.043*
	Mean ± SD	21.00 ± 7.92	16.00 ± 7.19	
1st month	Min-Max (Median)	6–42 (25)	10–30 (20,5)	^a^0.170
	Mean ± SD	24.06 ± 9.38	20.77 ± 5.25	
6th month	Min-Max (Median)	6–45 (25)	10–30 (22.5)	^a^0.211
	Mean ± SD	24.44 ± 10.21	21.23 ± 5.48	
	^b^ *p*	0.001**	0.001**	
Differences				
Fracture – Initial reduction	Mean ± SD (Median)	−11.11 ± 8.55 (− 11.5)	−14.82 ± 9.91 (− 13)	^d^0.215
	^c^ *p*	0.001**	0.001**	
Fracture – 1st month	Mean ± SD (Median)	−8.06 ± 8.52 (−10)	− 10.05 ± 8.21 (− 9,5)	^d^0.614
	^c^ *p*	0.005**	0.001**	
Fracture – 6th month	Mean ± SD (Median)	−7.67 ± 9.75 (−9,5)	− 9.59 ± 9.15 (−10)	^d^0.558
	^c^ *p*	0.023*	0.001**	
Initial reduction – 1st month	Mean ± SD (Median)	3.06 ± 4.87 (3)	4.77 ± 6.10 (5)	^d^0.195
	^c^ *p*	0.098	0.009**	
Initial reduction – 6th month	Mean ± SD (Median)	3.44 ± 6.25 (3)	5.23 ± 6.68 (5)	^d^0,270
	^c^ *p*	0.191	0.009**	
1st month – 6th month	Mean ± SD (Median)	0.39 ± 2.12 (0)	0.45 ± 2.06 (0)	^d^0.868
	^c^ *p*	1.000	1.000	

*UGS* Ulnar gutter splint, *(FMS)* Functional metacarpal splint, *Min* Minimum, *Max* Maximum, *SD* Standard deviation

*p < 0.05, **p < 0.01

^a^Student t test

^b^two-way ANOVA test

^c^Post Hoc: Bonferroni test

^d^Mann Whitney U test

When the metacarpal shortness of both groups was analyzed, the average shortening of the fractures were similar between the groups before reduction. After reduction, significant correction was achieved in both groups. (*p* = 0.001) The length could not be maintained between the initial reduction and the 1st month in both groups, whereas the average shortening values were similar between the 1st and 6th month follow-ups in both groups, and these findings are similar to the angulation measurements (Table 3).

**Table 3:** Evaluation of periodic metacarpal shortness measurements and differences according to the splint method

		UGS (*n* = 18)	FMS (*n* = 22)	^d^ *P*
Metacarpal shortening measurements (mm)				
Fracture	Min-Max (Median)	2–7 (5)	2–9 (4)	0.303
	Mean ± SD	4.67 ± 1.46	4.32 ± 1.46	
Initial reduction	Min-Max (Median)	1–6 (2)	1–4 (2)	0.209
	Mean ± SD	2.83 ± 1.58	2.14 ± 0.94	
1st month	Min-Max (Median)	1–7 (3.5)	2–5 (3)	0.522
	Mean ± SD	3.61 ± 1.69	3.27 ± 1.08	
6th month	Min-Max (Median)	1–6 (4)	2–5 (3)	0.359
	Mean ± SD	3.67 ± 1.71	3.32 ± 1.04	
	^e^ *p*	0.001**	0.001**	
Differences				
Fracture – Initial reduction	Mean ± SD (Median)	−1.83 ± 1.34 (− 1)	−2.18 ± 1.47 (− 2)	0.292
	^f^ *p*	0.001**	0.001**	
Fracture – 1st month	Mean ± SD (Median)	−1.06 ± 1.11 (− 1)	−1.05 ± 1.17 (− 1)	0.910
	^f^ *p*	0.002**	0.001**	
Fracture – 6th month	Mean ± SD (Median)	−1.00 ± 1.08 (− 1)	−1.00 ± 1.15 (− 1)	0.909
	^f^ *p*	0.002**	0.001**	
Initial reduction – 1st month	Mean ± SD (Median)	0.78 ± 1.26 (0,5)	1.14 ± 1.08 (1)	0.271
	^f^ *p*	0.018*	0.001**	
Initial reduction – 6th month	Mean ± SD (Median)	0.83 ± 1.34 (0,5)	1.18 ± 1.05 (1)	0.269
	^f^ *p*	0,018*	0,001**	
1st month – 6th month	Mean ± SD (Median)	0.06 ± 0.54 (0)	0.05 ± 0.21 (0)	0.895
	^f^ *p*	0.655	0.317	

*UGS* Ulnar gutter splint, *(FMS)* Functional metacarpal splint, *Min* Minimum, *Max* Maximum, *SD* Standard deviation

*p < 0.05, **p < 0.01

^d^Mann Whitney U Test

^e^Friedman Test

^f^Wilcoxon Signed Ranks Test

The *Quick*DASH scores of the two groups were similar at the 2nd and 6th month follow-ups. In the FMS group, the improvement in *Quick*DASH scores between the 2nd and 6th month follow-ups was significant (*p* = 0.003) but not in the UGS group (*p* = 0.075). When the amount of improvement between the 2nd and 6th months were compared according to the splint method, the difference was not statistically significant (Table 4).

**Table 4:** Evaluation of periodic *Quick*DASH score calculations and differences according to the splint method

		UGS (*n* = 18)	FMS (*n* = 22)	^a^ *P*
*Quick*DASH scores				
2nd month	Min-Max (Median)	0–36.3 (7.9)	0–34.1 (3.4)	0.760
	Mean ± SD	9.77 ± 11.16	7.22 ± 9.43	
6th month	Min-Max (Median)	0–40.9 (1.7)	0–15.9 (0)	0.179
	Mean ± SD	4.36 ± 9.53	2.01 ± 4.14	
	^c^ *p*	0.075	0.003**	
Differences				
2nd – 6th month	Mean ± SD (Median)	−5.41 ± 13.13 (− 1.7)	− 5.21 ± 7.43 (− 3.4)	0.846

*Quick*DASH The disabilities of the arm, shoulder and hand (DASH) score, *UGS* Ulnar gutter splint, *(FMS)* Functional metacarpal splint, *Min* Minimum, *Max* Maximum, *SD* Standard deviation

**p < 0.01

^a^Mann Whitney U Test

^c^Wilcoxon Signed Ranks Test

When the grip strengths were evaluated, the average grip strengths were similar between the groups for the 2 and 6th month evaluations. Both groups achieved remarkable improvement in grip strength between the 2nd and 6th months. (*p* = 0.001) When the expected grip strengths were calculated according to the healthy hand grip strength of the patients, the FMS group reached the expected strength values at the 2nd month follow-up, whereas the UGS group still exhibited significantly reduced grip strength at the 2nd month follow-up. (*p* = 0.008) However, at the end of 6th month follow-up, both groups had grip strength values similar to their expected grip strength values, which were calculated from the healthy hand grip strength of the patients (Table 5).

**Table 5:** Evaluation of periodic grip strength measurements and differences according to the splint method

Grip strength measurements (lbs)		UGS (*n* = 18)	FMS (*n* = 22)	^a^ *P*
Fractured side				
2nd month	Min-Max (Median)	49–107 (80)	40–106 (73)	^a^0.608
	Mean ± SD	77.06 ± 16.16	74.32 ± 17.03	
6th month	Min-Max (Median)	53–120 (92,5)	55–112 (82,5)	^a^0.498
	Mean ± SD	89.67 ± 18.97	85.95 ± 15.36	
	^c^ *p*	0.001**	0,001**	
2nd - 6th month increase	Mean ± SD (Median)	17.08 ± 15.47 (16.2)	17.57 ± 13.37 (15.1)	^a^0.892
Loss of grip strength (lbs)				
2nd month	Mean ± SD (Median)	14.12 ± 20.89 (10.6)	6.46 ± 16.28 (1.3)	^a^0.226
	^c^ *p*	0.008**	0.097	
6th month	Mean ± SD (Median)	0.84 ± 13.49 (− 4.1)	−2.24 ± 16.58 (−4.4)	^a^0.673
	^c^ *p*	0.982	0.383	

*UGS* Ulnar gutter splint, *(FMS)* Functional metacarpal splint, *Min* Minimum, *Max* Maximum, *SD* Standard deviation

*p < 0.05, **p < 0.01

^a^Mann Whitney U Test

^c^Wilcoxon Signed Ranks Test

## Discussion

The objective of this study was to compare the maintenance of the reduction, functional outcomes and grip strength of adult patients who were treated with FMS or UGS for closed fifth metacarpal neck fractures. FMS provided faster improvement in clinical scores and earlier gain of normal grip strength compared to UGS with similar reduction maintenance. FMS does not limit the wrist motion or MCP joints and is easier to be performed by the physicians compared with UGS, which is more bulky and limits both wrist and MCP movements. Therefore, it is possible to say the patient adherence to FMS might be better due to increased patient comfort.

It is important to maintain the reduction in metacarpal fractures to avoid the reduced grip strength and pseudoclawing at the MCP joint caused by excessive volar angulation [11, 13]. The studies by Konradsen et al. and Jones reported improvement in fracture angulation with a functional cast, which allowed free motion of the wrist and finger joints, to treat metacarpal shaft and neck fractures [23, 24]. Debnath et al. also demonstrated the effectiveness of Barton’s short hand cast in maintaining angulation for reduced metacarpal shaft fractures [9]. However, none of these studies evaluated the functional outcomes of the patients. Kuokkanen et al. compared splinting after reduction and functional bandage without reduction in the treatment of 5th metacarpal neck fractures. They found that angulation generally remained at the same level in both groups throughout the follow-ups. Despite similar functional results at the 3-month follow-up, grip strength and range of motion were better in the bandage group at 4 weeks. The elastic bandage group had an average 42 degrees (range, 20–60) angulation, whereas the reduction/splinting group had an average 30 degrees (range, 10–50) angulation. High displacement values in the reduction/splinting group makes it difficult to observe possible benefits of a good reduction compared with the elastic bandage group. They also did not use a validated outcome measure, preventing the evaluation of true functionality [25].

Similar to angulation, excessive shortening was also thought to be responsible for functional impairment at the MCP joint by an anatomical study [26]. However, its clinical relevance is not sufficiently reviewed, and Lumplesch et al. reported no extension deficit for shortening up to 6 mm [27]. Aaken et al. also reported no extension lag with shortening of up to 5 mm [28]. In our study, after initial reduction and splint application, the angulation measurements were better in patients treated with FMS. The difficulty in applying UGS potentially prevented the maintenance of the initial reduction during the splint application. However, at the 1st month follow-up, reduction loss was more significant in the FMS group compared with the UGS group. At the 1st and 6th month follow-up, average angulation values were similar between groups and in between the acceptable range. Therefore, better reduction capacity of FMS at initial application could not be maintained throughout the follow-ups and did not provide any advantage for improved reduction compared with the UGS method. Both methods resulted in similar angulation and shortening values at the end of the 6th month follow-up. Maintenance of the angulation and length of the 5th metacarpal between the 1st and 6th month follow-ups in both groups reveals that adequate healing was achieved at the 1st month, and splint usage is no longer needed.

The clinical outcomes of the treatment methods can be reported in numerous ways like satisfaction rates, return to work percentages and patient reported outcome measurements. Muller et al. compared the satisfaction rates and the return to work of the patients treated with cast or bandage and they didn’t detect any significant difference [8]. As a validated hand outcome measurement, *Quick*DASH has been used to discern disabilities from musculoskeletal disorders of the upper limb and consists of three subscales including disability/symptom, and work and sports/performing arts [18, 19, 29]. *Quick*DASH was used in numerous studies to evaluate the treatment results for hand injuries but the reports with *Quick*DASH measurements of metacarpal injuries are few [15, 28, 30, 31]. Hofmeister et al. compared two different casting methods in the treatment of fifth metacarpal neck fractures and they didn’t detect any difference between the DASH scores. In our study, *Quick*DASH score improvement between 2nd and 6th month follow-up was more significant in the FMS group, indicating that FMS might be related to better functional improvement. However, the average *Quick*DASH scores at the end of 6th month follow-up were similar in both groups, demonstrating that similar and satisfying functional outcomes were achieved with both methods.

Early regain of grip strength is an important parameter, especially for manual laborers. In a study by Davison et al., hand-based thermoplastic splints resulted in improved early range of motion and grip strength in pediatric patients compared with UGS in the treatment of 5th metacarpal neck fractures [32]. In our study, despite similar 6th month follow-up values, the patients treated with FMS exhibited earlier regain of the normal grip strength.

At present, there is no consensus on the optimal management of 5th metacarpal neck fractures. Advantages of the FMS treatment method include its efficacy, ease of application, freedom of wrist and IP joint motion, early regain of grip strength, satisfactory functional scores and improved patient tolerance. Active and passive motion of the distal interphalangeal and proximal interphalangeal joints of the ring and small fingers are allowed, therefore permitting daily activities, such as eating, writing, and grasping.

This study has several limitations. Although the study design was prospective, it was not randomized or controlled. Eighteen patients (31%) did not return for routine evaluations and were lost to follow-up. We think the most probable reason behind this high rate is the great healing and functional capacity of 5th metacarpal neck fractures especially in younger population. The study group didn’t include patients older than 58 years old, so it is not possible to generalize our results to elder population. Possible intraobserver variability for radiological and functional measurements might also be considered as another limitation.

## Conclusions

Despite the greater reduction loss at 1st month, FMS is adequate in the maintenance of acceptable reduction and may provide faster improvement in clinical scores and earlier gain of normal grip strength compared with UGS. However, at the 6th month follow-up, no superiority was detected between FMS and UGS methods. FMS restricts less joints and provides better functionality compared with UGS, and this information should be considered when deciding the treatment method.

## Abbreviations

AP: Anteroposterior
DASH: Disabilities of the Arm, Shoulder and Hand
FMS: Functional metacarpal splint
MCP: Metacarpophalangeal
PIP: Proximal interphalangeal
SD: Standard deviation
SH-Stip: Shortening stipulated
UGS: Ulnar gutter splint
